# The Inhalation of Steam in Diseases of the Mucous Membrane

**Published:** 1853-07

**Authors:** Edward Govett


*The Inhalation of Steam in Diseases of the Mucous Membrane.* By Edward Govett, M. R., C. S. L.—Having had many opportunities of testing its utility (personally as well as upon my patients), perhaps a few observations upon the subject may be found useful.

I have known the action of steam upon the mucous membrane of the air-passages, when in a state of spasm or inflammation, in some cases immediately beneficial, particularly in croup (from which disease I had when a child suffered most severely and repeatedly), also in hooping-cough and asthma. In the latter disease I have constantly seen almost instant relief, particularly in a case under my care during my homeward winter passage from Australia round Cape Horn, where the temperature was extremely low, and the winds most violent and keen, accompanied with constant wet and dampness—a state of things least calculated for the well-doing of a case of this kind. The paroxysms of coughing in one patient were violent and distressing in the extreme until he had recourse to the vapor of boiling or hot water, when the symptoms immediately began to relax, and the fit soon terminated. Such indeed was the beneficial effect of the inhalation that the poor sufferer constantly kept his "pannikin" within reach, that he might be instantly supplied with water from the galley. It is needless to say that this, unlike most other remedies for internal diseases, is brought into immediate contact with the part affected, and appears most unquestionably to possess the power of relaxing spasms and allaying irritation, an object of immense importance in the three above-named diseases.

I have also every reason to believe that great benefit would result from the adoption of this treatment in cases of pneumonia and even pleuritis. I cannot, however, adduce the result of any experience of its application in the two latter forms of disease, having as yet never extended its employment to them, but which I certainly intend doing upon the first opportunity.

The vessel I usually recommend for the purpose is a common earthen or metal teapot, and if the opening be smaller than the general circumference so much the better, as the steam then ascends in a more compact or concentrated body. A piece of flannel should be placed around the edges that the face may not be scalded by contact with the hot metal or ware; the mouth and nose are to be kept closely and constantly applied over the opening, leaving only sufficient room for the admission of atmospheric air.—*Ibid.*

